# Monitoring and Follow-Up of Patients on Vitamin K Antagonist Oral Anticoagulant Therapy Using Artificial Intelligence: The AIto-Control Project

**DOI:** 10.3390/jcm14207191

**Published:** 2025-10-12

**Authors:** Adolfo Romero-Arana, Nerea Romero-Sibajas, Elena Arroyo-Bello, Adolfo Romero-Ruiz, Juan Gómez-Salgado

**Affiliations:** 1 Fundación Jiménez Díaz School of Nursing, Health Research Institute, Fundación Jiménez Díaz University Hospital, Universidad Autónoma de Madrid (IIS-FJD, UAM), 28040 Madrid, Spain; adolfo.romero@quironsalud.es (A.R.-A.); elena.arroyo@quironsalud.es (E.A.-B.); 2 La Paz University Hospital, Madrid Health Service, 28046 Madrid, Spain; nerearomero95@gmail.com; 3 BA-16 Group, Institute of Biomedical Research of Malaga and Platform in Nanomedicine-IBIMA Platform BIONAND, 29590 Málaga, Spain; 4 Research Unit, Hospital Virgen de la Victoria, Andalusian Health Service, 29010 Málaga, Spain; 5 Department of Nursing and Podiatry, Health Sciences School, University of Málaga, 29071 Málaga, Spain; 6 Department of Sociology, Social Work and Public Health, Faculty of Labour Sciences, University of Huelva, 21007 Huelva, Spain; salgado@uhu.es; 7 Safety and Health Postgraduate Programme, Universidad Espíritu Santo, Guayaquil 092301, Ecuador

**Keywords:** anticoagulation management program, vitamin K antagonist, app development, AI assistants

## Abstract

**Background**: Vitamin K antagonist oral anticoagulant (VKA) therapy, using warfarin or acenocoumarol in our health system, is indicated, according to clinical guidelines, for the prophylaxis of thromboembolic events. In Málaga, the VKA patient management program currently includes a total of 856 patients. **Hypothesis**: The use of an AI-based application can enhance treatment adherence among VKA patients participating in self-monitoring and self-management programs. Furthermore, it can support the comprehensive implementation of the system, leading to reduced costs and fewer interventions for anticoagulated patients. **Methods**: The study will be conducted in several phases. The first phase involves the development of the application and the integration of Artificial Intelligence (AI) and Machine Learning (ML) algorithms. The second phase includes preliminary testing and validation of the developed application. The third phase consists of full implementation, along with an assessment of user-identified needs and potential quality improvements. **Expected Results**: The implementation of the *AIto-Control* app is expected to reduce healthcare-related costs by decreasing primary care visits and hospital admissions due to thromboembolic or bleeding events. Additionally, it aims to ease the workload on both primary care and hospital services. These outcomes will be achieved through the involvement of advanced practice nurses who will supervise app-based monitoring and patient education.

## 1. Introduction

Vitamin K antagonist oral anticoagulant (VKA) therapy, using warfarin or acenocoumarol in our health system, is indicated, according to technical guidelines, to prevent thromboembolic events. These adverse events occur after a venous thromboembolic episode or under specific circumstances, such as atrial fibrillation, thrombophilia, or valve replacement [1].

The main indication for oral anticoagulant therapy is atrial fibrillation. This chronic cardiac arrhythmia is the most frequently described in clinical practice, with a prevalence in the general population of approximately 1–2%, although this prevalence is strongly associated with age. In Spain, the prevalence of atrial fibrillation is 4.4%, with a similar distribution between sexes and an increase after 60 years of age [2]. Patients with atrial fibrillation are at greater risk of embolic events, the most frequent being stroke, which is associated with disability, dependency, and reduced quality of life, leading to a significant increase in healthcare costs and resource utilization [2].

Without treatment, the risk of stroke and other comorbidities is high. In fact, atrial fibrillation increases the risk of stroke fivefold, accounting for one in every five cases of cardioembolic stroke. The risk rises with age, as the prevalence of stroke in individuals older than 80 years in Spain reaches 20% [2]. Similarly, ischemic stroke associated with atrial fibrillation involves a higher fatality rate, doubling the risk of death compared to stroke unrelated to atrial fibrillation. Moreover, survivors often experience a high degree of disability, which is the main reason for recurrence [1,3,4].

Atrial fibrillation patients represent up to 60% of individuals monitored in primary care centers or anticoagulation clinics for oral anticoagulant therapy control or follow-up [5,6], although this proportion depends greatly on the region of residence. In recent years, direct oral anticoagulants have been increasingly used for this condition, provided there is no underlying valvular disease [7,8,9,10].

The next most frequent indication for VKA treatment is the prevention of venous thromboembolic events for several months in primary cases, followed by chronic therapy in recurrent cases [11]. VKA is also required for cardiac valve prostheses, particularly mechanical ones, and certain fibrinolysis disorders [12].

Population aging has increased the demand for healthcare resources and services, both due to the higher incidence of chronic diseases and longer life expectancy. In this context, the ability of patients to manage their symptoms appropriately through effective self-care becomes increasingly relevant [5]. As noted earlier, there is a strong correlation between age and atrial fibrillation, since the proportion of patients older than 80 years can reach 19% [2]. These patients usually initiate anticoagulant therapy in a hospital anticoagulation office, and once stabilized, they are referred to their primary care centers.

In many settings, nurses are responsible for dosage adjustment and follow-up, following regional and national protocols [13], in collaboration with hematologists in a hospital anticoagulation office and primary care physicians in primary care centers. This model seeks to ensure continuity of care and improve the management of healthcare resources. However, it is common for patients returning to a hospital anticoagulation office to report doubts, lack of knowledge, and adherence issues, which may require more reliable tools to assess adherence and knowledge levels, as well as additional educational support [6].

Another relevant aspect of this discussion is the assessment of self-care ability and treatment adherence using validated instruments, which is essential for patients requiring strict therapeutic compliance to ensure efficacy and safety. The objective is to apply a scale to identify individuals with inadequate self-management, or, in more contemporary terms, to determine their readiness for enhanced self-care (NANDA-I Classification, NANDA International, 2024–26, Nursing Diagnosis) among chronic patients. The DecaMIRT scale has demonstrated that the risk of hospital readmission is four times higher in patients with poor treatment adherence and that quality of life declines by more than five points per year in patients with COPD [14]. DecaMIRT has shown validity and utility in chronic patients, such as those on anticoagulant therapy, and could be useful for detecting adherence problems in this population. This approach allows for the use of Nursing Outcomes Classification (NOC) results as indicators of treatment compliance. Additionally, in our healthcare system, the DecaMIRT scale will be integrated into the Andalusian health service web applications to assess treatment adherence.

In recent years, the introduction of portable anticoagulometers has enabled the follow-up of anticoagulated patients at home (self-monitoring) and, in some cases, dosage adjustment based on obtained results (self-management) [4]. These devices employ dry-chemistry methods to analyze a few drops of capillary blood and obtain prothrombin times comparable to laboratory values. Various models with similar performance are available, allowing for accurate results based on the international normalized ratio (INR) [15].

In Málaga, the VKA patient management program includes 856 patients distributed across several health areas belonging to two reference hospitals: Virgen de la Victoria Hospital, with an anticoagulation office in Valle del Guadalhorce Hospital, following 351 patients, and the Regional de Málaga Hospital, with an office in Materno Infantil Hospital, following 495 patients.

This program enables patients to adjust their medication according to INR values in two ways. First, patients may consult a healthcare professional who indicates the appropriate dose at each moment, a modality known as self-analysis or self-monitoring [4,15]. The second method involves following a predefined algorithm for dosage adjustment, known as self-management. Both approaches have demonstrated efficacy and cost-effectiveness, even when compared with direct oral anticoagulants [10]. This allows patients to maintain better therapeutic control, achieving improved Rosendaal Time in Therapeutic Range (TTR) outcomes [16].

Unfortunately, access to this type of follow-up requires meeting certain criteria that many anticoagulated patients do not fulfill, mainly due to advanced age and low educational levels [17,18]. Consequently, they often face difficulties completing the required training. According to a study conducted by the Federación Española de Asociaciones de Anticoagulados (FEASAN) in 2013, 65% of patients were older than 65 years, and 44% had only primary education [19]. Maintaining good adherence to the therapeutic regimen is a critical factor for success; indeed, a study demonstrated that adherence to treatment correlates strongly with adherence to warfarin therapy, highlighting the importance of educational and motivational strategies for improving compliance [20].

The implementation of an artificial intelligence (AI)-based dosing support system could help incorporate these patients into this therapeutic modality. In 2017, a study showed that, although with a limited sample size, patients improved their adherence with this type of support, not only those anticoagulated with VKA but also those receiving direct oral anticoagulants [21]. Given the major advancements in AI development over the past eight years, current results could be even more promising. The combination of AI and pharmacogenomics shows potential for precision-guided, real-time anticoagulant therapy [22].

The progress of AI in recent years has enabled significant improvements in patient follow-up, particularly in cardiovascular care [23] and specifically through the development of digital applications [24]. Consequently, the creation of machine learning (ML) models has become increasingly important, such as those developed in Korea for warfarin dosing [25]. The use of ML has been successfully implemented over the past decade in both hospital and primary care settings for purposes including dosage adjustment, patient monitoring, and economic forecasting [25].

## 2. Hypotheses and Aims

### 2.1. Hypotheses

The use of an AI-based application can improve treatment adherence in VKA patients enrolled in self-monitoring and self-management anticoagulation programs and supports the full implementation of the system, thereby reducing healthcare costs and unnecessary interventions for anticoagulated patients.

### 2.2. Aims

#### 2.2.1. Main Aim

The main aim of this study is to develop an AI-based application (app) to monitor, control, and follow-up patients treated with vitamin K antagonist oral anticoagulant therapy.

#### 2.2.2. Secondary Aims

The following are the secondary aims of this research:

- To validate an AI and ML tool in the anticoagulation context.
- To evaluate the use of the AIto-Control app versus normal follow-up for VKA patients in both hospital and primary care centers.
- To explore the needs and expectations of VKA patients in self-monitoring and self-management programs.
- To evaluate the levels of satisfaction and usability from patients that use the AIto-Control app.

## 3. Materials and Methods

The study will be conducted in several phases. The first phase will involve the development of the application and the integration of AI and ML components. The second phase will consist of preliminary testing and validation of the developed application. The third phase will focus on its full implementation, including an assessment of user-identified needs and quality improvement measures.

The study will involve several participating centers across the Málaga region. The main facilities include the Virgen de la Victoria University Hospital and Málaga Regional University Hospital. Additionally, centers from two health districts will participate: the Costa del Sol Health District, represented by the San Pedro Alcántara Health Centre, and the Málaga–Guadalhorce Health District, which will contribute through both the Churriana Health Centre and the El Cónsul Health Centre.

### 3.1. Inclusion and Exclusion Criteria

#### 3.1.1. First Phase (Feeding Phase)

The inclusion criteria for this phase prioritize patients included in the TAONet database, aged over 18 years, who gave their consent for their medical records to be consulted.

#### 3.1.2. Second Phase

The inclusion criteria for this phase will prioritize patients able to identify potential technical errors and proficiency in the use of mobile technologies. However, patients lacking these skills may also participate if they have a primary caregiver capable of handling the app. All participants, patients, or caregivers, will receive a 30 min guided tutorial on app functionalities and troubleshooting.

This initial recruitment of young, technologically skilled participants corresponds to a pilot usability phase aimed at detecting technical errors and optimizing app usability. Subsequently, the inclusion criteria will be broadened to elderly and low-literacy patients, with caregiver assistance where necessary, ensuring full representativeness of the VKA population.

#### 3.1.3. Third Phase (Quasi-Experimental Study/Qualitative Phase)

##### Inclusion Criteria

The inclusion criteria for this phase prioritize patients from participating units aged 18 years or older, with no upper age limit, who can understand and use mobile applications, or who have a primary caregiver able to do so on their behalf.

##### Exclusion Criteria

The exclusion criteria include the following:

- Withdrawal from the follow-up program for any reason.
- Transition to a direct-acting oral anticoagulant.
- Change of residence to a healthcare area outside the coverage of our service.

Patients who die during the study period will not be excluded; deaths will be treated as censored observations in the analysis, with causes of death (e.g., thrombotic or bleeding events) recorded for outcome assessment.

### 3.2. Procedures

#### 3.2.1. Phase 1

**Figure 1:**
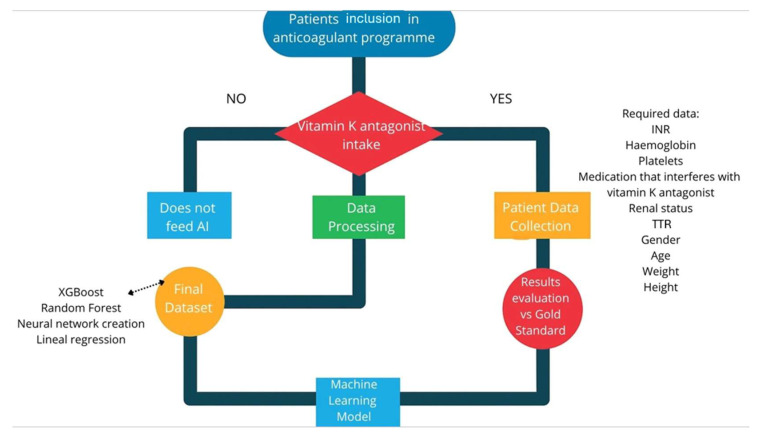
Machine learning diagram (self-elaboration).

In this phase, a descriptive cross-sectional study will be conducted. Data will be obtained from patients registered in the TAONet^®^ web application, developed by Roche Diagnostics (Basel, Switzerland) and integrated into the Andalusian Health System. Data processing will be performed using an ML model (Figure 1). The dataset will include records from patients registered over the past five years (2020–2025).

Once the data has been collected, a comparison will be made between the monitoring provided by the AI-powered system and the current gold standard, which consists of TAONet monitoring combined with coagulometer measurements. Data will be gathered by nurses working in the anticoagulation offices at Hospital Regional and Virgen de la Victoria, both located in Málaga. Laboratory INR values will also be considered when deemed clinically necessary. As this is an observational study, the values analyzed through ML will not be used for therapeutic decision-making at this initial stage.

The ML training dataset will include INR data obtained from TAONet. All variables represented in Figure 1 are available in each patient’s TAONet profile or can be retrieved from their electronic health records. The ML model will be developed using Jamovi 2.7.6, employing modules such as SnowCluster. The full data acquisition protocol will be published once the model is completed, following the STARD-AI checklist.

Model features include the six-month INR history, current vitamin K antagonist oral anticoagulant dose, comorbidities, interacting medications, age, and weight. The model will be trained with 80% of the TAONet data from the period 2020–2024, with 20% reserved for validation, while data from 2025–2026 will be used for external testing to assess real-world performance.

Feature selection will be performed using recursive feature elimination based on the model’s performance on validation data. The ensemble method will combine gradient boosting and random forest algorithms to enhance predictive accuracy and reduce overfitting. Model performance will be reported according to the STARD-AI checklist, including AUC, sensitivity, specificity, and calibration plots.

After establishing the ML workflow, a mobile web application will be developed for patient monitoring. The application will be built using the health artificial intelligence model, part of ADA Artificial Intelligence, a system specialized in healthcare knowledge. Its integration will provide a dynamic monitoring tool for anticoagulated patients. The Health by ADA model has been validated in various studies in recent years [26,27,28]. A stacking ensemble method will be implemented, combining XGBoost, Random Forest, and linear regression as base models, with logistic regression serving as the meta-learner to integrate predictions.

The application will allow for customization of health, diagnostic, dietary, TTR, and INR dosage data, enabling patients to monitor their own treatment. All data generated will be stored on a secure native platform, supervised by nursing staff to assist patients with questions or to address errors related to dosage or health recommendations.

The backend will be developed in Python 3.13, selected for its simplicity, readability, and suitability for healthcare applications, where precision and transparency are crucial. Clear and maintainable code enhances team collaboration and facilitates rapid modifications when required. Python also offers a wide range of libraries and frameworks, such as TensorFlow, PyTorch 2.8, and scikit-learn, which support the efficient implementation of ML models essential for processing and analyzing patient information. These capabilities allow for a full integration of ADA’s AI functionalities to deliver accurate and personalized monitoring.

The frontend will be developed using React Native with TypeScript, a combination that enables the creation of a single code base for both iOS and Android platforms, thereby reducing development time and costs without compromising performance. Python’s scalability also ensures that the system can expand as the number of patients and functional requirements increase.

TypeScript and React Native, both based on JavaScript, are used for frontend development.

React Native allows for the following:

- The creation of native interfaces from a single code base.
- Access to device features, such as the camera and GPS.
- Optimal performance through its native bridge.
- Simplified application updates and maintenance.

TypeScript provides the following:

- Static typing to prevent runtime errors.
- Greater code clarity and organization.
- Enhanced autocompletion and real-time error detection.
- Improved project scalability and maintainability.

**Figure 2:**
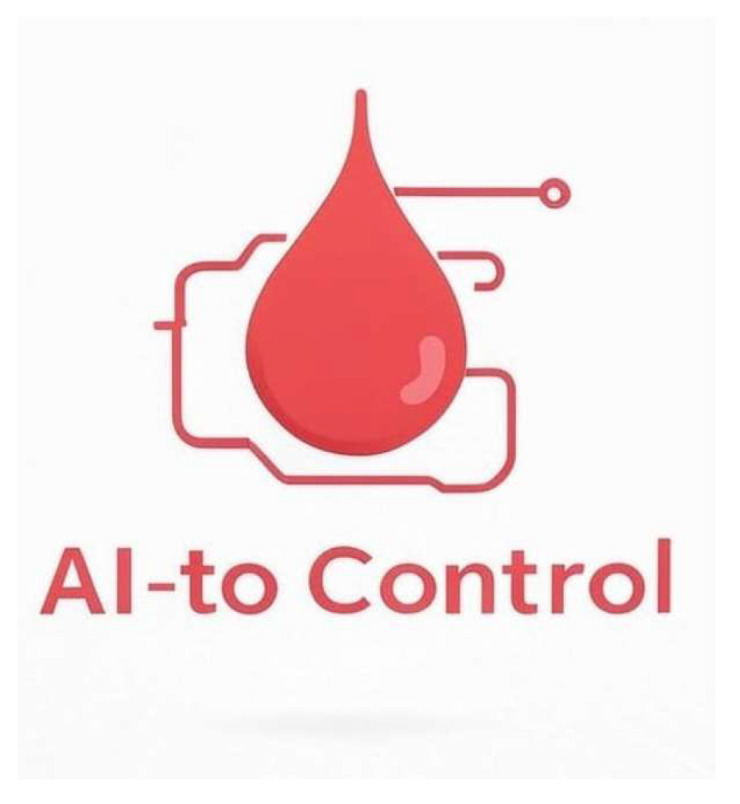
App logo (self-elaboration).

**Figure 3:**
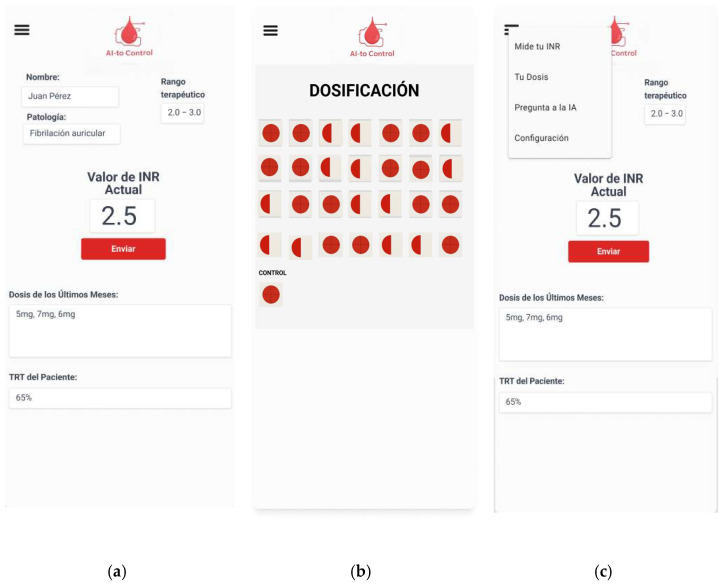
App menus. From left to right: (**a**) main page; (**b**) dosage screen; (**c**) menu (self-elaboration).

The prototype and initial interface designs of the application (Figure 2 and Figure 3) were created using FIGMA, prioritizing a clean and intuitive layout that facilitates use by patients, including those with limited familiarity with digital technologies.

Furthermore, since the application will handle data related to patients’ medical records, access will be secured through biometric authentication, as well as through the Cl@ve system or a digital certificate.

#### 3.2.2. Phase 2

The second phase will correspond to a quasi-experimental study, divided into several subphases for the implementation of the developed application.

The first subphase will involve a preliminary pilot study to test the app. Patients enrolled in the self-monitoring programs at the Virgen de la Victoria and Regional University Hospitals of Málaga will be selected. The total sample in the self-monitoring groups from both hospitals comprises 846 patients. To ensure a closer and more personalized follow-up and to detect potential errors in the application, the sample will be selected based on the following criteria, in accordance with previous studies conducted by our team [29]:

- Aged ≥ 18 years: Although the pilot phase focuses on younger participants for error detection and usability testing, later phases (Phase 3) will include elderly and low-literacy patients, ensuring representativeness of the real VKA population and improving external validity.
- Receiving treatment with acenocoumarol or warfarin.

The patients included will monitor themselves using both the app and their usual self-monitoring program to avoid compromising their healthcare. A total of 145 patients will be included in this first subphase. The sample size for Phase 2 was calculated using G*Power 3.1, assuming a 3-point improvement in TTR [29], with α = 0.05, power = 80%, and 10% attrition, resulting in a required sample size of 145 patients per group.

The control group will consist of the remaining patients in the anticoagulated patient self-monitoring program. In addition, 145 patients on stable VKA therapy will be included to validate the app’s INR measurement accuracy against TAONet/coagulometer results. These patients will have their INR monitored exclusively through the application to subsequently evaluate its internal and external validity.

Thus, the total sample for Phase 2 will comprise 290 participants, with 145 assigned to the AIto-Control group and 145 to the control group following usual care.

Two modes of patient participation will be possible: first, patients may perform self-monitoring, measuring INR at home and consulting a health professional for dose adjustments; second, patients may perform self-management, measure INR, and adjust the dose themselves according to a predefined algorithm [17].

This testing and trial phase of the application is expected to last six months. Once the use of the app has been positively evaluated and any quality-of-life improvements suggested by the first group have been implemented, it will be progressively rolled out to the rest of the self-monitoring patients.

This phased implementation strategy will prevent data server overload, which could interfere with the monitoring and management of anticoagulated patients. To ensure system stability, user activation will occur gradually, at a rate of approximately 100–126 patients per month, achieving full implementation over a period of six to seven months.

#### 3.2.3. Phase 3

The final phase will correspond to the concluding stage of the quasi-experimental study and the beginning of the qualitative component. A mixed-methods approach will be applied, including several focus groups and a SWOT (Strengths, Weaknesses, Opportunities, and Threats) analysis. These activities will help identify patients’ needs and expectations regarding the use, optimization, and potential quality-of-life improvements associated with the application.

The control group will include patients receiving anticoagulation therapy with vitamin K antagonists who are monitored in both primary care and hospital settings. The sample will mirror that of the self-monitoring group, comprising 846 patients. Following this stage, full implementation of the application will be proposed for all patients treated with vitamin K antagonists, and a formal proposal will be submitted to the Andalusian Health Service after the registration of the app as a utility model.

The qualitative component will include the development of focus groups and the execution of a SWOT analysis to identify the strengths, weaknesses, opportunities, and threats related to the program’s implementation.

### 3.3. Study Variables

The study variables are as follow (Table 1).

### 3.4. Data Analysis

To evaluate compliance with the study objectives, an initial descriptive analysis will be performed. Measures of central tendency and dispersion (means and standard deviations for normally distributed variables and medians and quartiles for non-normally distributed ones) will be calculated for quantitative variables, while categorical variables will be summarized as frequencies and percentages.

To compare INR differences between the app group and the control group, Student’s *t*-test for independent samples (for normally distributed data) or the Mann–Whitney U test (for non-normal data) will be performed. Normality will be verified using the Kolmogorov–Smirnov, Shapiro–Wilk, and Levene tests. Logistic regression models will be constructed to identify predictors of treatment adherence.

Cluster analysis will also be performed using Ward’s method to obtain hierarchical clusters. This technique groups observations into relatively homogeneous categories by minimizing within-group variance. Ward’s method assumes that each data point initially forms its own cluster, then iteratively merges those that produce the smallest increase in total within-cluster variance, based on the Euclidean distance. Variables requiring visual exploration will be represented graphically to enhance interpretability. Clustering will allow for a more detailed understanding of differences by age, gender, and type of vitamin K antagonist, thus facilitating comparisons across social and demographic groups.

A multivariate logistic regression model will also be developed, incorporating the parameters SF-12, DECAMIrt, knowledge questionnaire, and TTR as explanatory factors.

To assess the validity of the application, its results will be compared with a reference standard (gold standard). Internal validity will be evaluated by calculating sensitivity, specificity, and the diagnostic likelihood ratio, while external validity will be determined using positive and negative predictive values.

To evaluate reliability, interobserver agreement will first be assessed by having each researcher re-evaluate de-identified INR datasets one week after the initial assessment to prevent memory bias. Researchers will compare the results of the same diagnostic test, specifically the INR values obtained by the patient and the dosages recommended by the application. Intraobserver agreement will be assessed by having each researcher re-evaluate INR datasets one week after the initial assessment, using de-identified and blinded datasets to prevent memory or selection bias. This will facilitate distribution of the data and enable multiple within-group comparisons. Agreement between methods will finally be assessed by comparing app-generated results with those of the reference standard.

Internal validity will be established through the calculation of sensitivity (the probability of obtaining a positive result in diseased individuals), specificity (the probability of obtaining a negative result in healthy individuals), and the likelihood ratio (the ratio between these probabilities).

External validity will be assessed through the positive predictive value (the probability of disease given a positive test result) and the negative predictive value (the probability of being healthy given a negative test result). In this study, positive results will correspond to INR values consistent with the laboratory results, whereas negative predictive values will refer to INR values obtained from patients on stable VKA therapy.

### 3.5. Qualitative Phase of the Study

At least one focus group will be conducted in each of the participating centers, resulting in an estimated sample size of approximately 40 participants. The principle of saturation will be applied to determine the final sample size, meaning that recruitment will continue until no significant new information emerges from the data [30]. Data saturation will be defined following Urquhart as “the point in coding when you find that no new codes occur in the data. There are mounting instances of the same codes, but no new ones” [31], a definition consistent with the principles of Grounded Theory.

Focus groups will continue until no new themes emerge across three consecutive sessions, following the principle of thematic saturation. An initial target of six to eight focus groups (each comprising four to six participants) is planned, with the final sample size determined by data saturation.

The sample will be selected intentionally, based on segmentation criteria designed to capture differences in perceptions during follow-up. Each focus group will include a leader or coordinator, a member of the research team, and between four and six patients.

Participants will be recruited according to the specifications outlined above and invited to attend the session, during which they will receive detailed information about the study and provide written informed consent.

The group coordinator will lead the session using a structured script developed by the research team. Information saturation will serve as the criterion for transitioning between topics and ultimately concluding the session. The script will be guided by participants’ needs and perceptions, while allowing for the emergence of additional themes beyond those initially anticipated.

An eidetic phenomenological approach will be employed to analyze the data, enabling the study of the phenomenon within its natural context and capturing participants’ experiences directly. This approach is particularly relevant in healthcare research, where emotions, perceptions, and personal meanings strongly influence patient needs, expectations, and demands [32].

A thematic analysis will be performed on the focus group data to facilitate understanding of the phenomena under investigation, including participants’ experiences and perspectives, as well as for analysis in other areas [33].

Additionally, Guba and Lincoln’s reliability criteria of credibility, dependability, transferability, and confirmability will be applied to ensure the scientific rigor of the study [34].

Finally, a SWOT analysis will be conducted to obtain a comprehensive understanding of the application’s usefulness for anticoagulated patients and to identify potential areas for improvement.

### 3.6. Ethical and Legal Aspects

This project is intended as a health research study and will therefore adhere to the recommendations of the Declaration of Helsinki, in its most recent edition (Brazil, 2013). It will also comply with all applicable ethical and legal requirements in our country, including Law 41/2002 on Patient Autonomy; Laws 15/1999 and 3/2018 on the Protection of Personal Data; as well as national and regional regulations governing research, such as Law 14/2007 and Order SAS/3470/2009 of 16 December, which establishes guidelines for post-authorization observational studies of medicinal products for human use. At no point will the study involve prescribing medication; only monitoring and dose adjustments according to the protocol approved by the Regional Ministry of Health will be performed.

The study procedures will be clearly explained in each session. For elderly participants or those with limited literacy, information will be provided both to the patients and their primary caregivers.

All data will be anonymized and securely stored on a server managed by the Andalusian Health System. Personal identifiers will not be included, and participant names will be replaced with unique codes in the format AICONTROL_XXX, with the code key stored in a separate encrypted database accessible only to the study’s data manager. Data transmission between the application and server will be secured using Proton VPN services, which provide unlimited storage and end-to-end encryption (E2EE). All data processing complies with GDPR Article 9 (special category data) and has been approved by the Andalusian Data Protection Authority.

## 4. Expected Results

Once the feasibility of implementing the AIto-Control application for monitoring patients on anticoagulant therapy with vitamin K antagonists has been established, a monitoring model will be proposed through the creation of an Advanced Practice Nurse (APN) in oral anticoagulation. The role of the APN is well established in nursing, encompassing scientific and professional responsibilities in a specific area of expertise.

This knowledge-based role, already implemented in areas such as chronic wound care, is highly relevant to the management of anticoagulated patients. The development of oral anticoagulation clinics by nursing staff in recent years provides an ideal foundation for the establishment of APNs in oral anticoagulation. Combined with the AIto-Control application, the APN will be able to assume a consulting and supportive role in the follow-up of anticoagulated patients, as well as providing support to the primary care network. Through the self-monitoring program using the AIto-Control app, the APN will provide guidance on diet, prescribed and additional medications, health education, and patient safety. For example, if a patient’s INR is elevated, the APN will serve as a liaison between the hematologist and the patient and coordinate administration of vitamin K if required.

The APN’s involvement in the scientific field will also facilitate improvements in the implementation of the application, as well as advancing care and scientific knowledge regarding patients anticoagulated with vitamin K antagonists.

Implementing this APN model will enhance organization and resource management, thereby improving healthcare services. The care of anticoagulated patients in both hospital and primary care settings generates significant patient flow and related healthcare expenditures. The estimated cost savings are theoretical, derived from current unit costs in Andalusian primary care and hospital services. A formal cost-effectiveness analysis will be conducted in future implementation phases to empirically validate these projections. In Andalusian primary care centers, scheduled consultations cost EUR 53.51, while home visits increase the cost to EUR 160.52. When considering hospital stroke-related costs (ranging from EUR 4279.80 to EUR 14,027.83 for ischemic stroke or EUR 4777.43 to EUR 12,408.06 for hemorrhagic stroke) and hospital appointment costs (EUR 110.71 for the first appointment, EUR 55.36 for follow-up), the estimated annual savings could be substantial. For example, follow-up of the 846 anticoagulated patients in Málaga could save approximately EUR 562,014.72 per year (EUR 55.36 per appointment, 12 months).

Based on previous studies conducted by our team [29], we expect an improvement in TTR equal to or greater than that observed in prior work, corresponding to an increase of at least three points in TTR.

Therefore, we propose the implementation of an APN care model that will benefit all areas of our health service: first, by enabling direct clinical application with home monitoring; second, by promoting technological development and innovation through artificial intelligence; third, by improving organization and resource management, thereby reducing waiting lists and alleviating pressure in hospitals and primary care; and finally, by contributing to broader improvements in healthcare services and policies, providing economic, managerial, and resource-based solutions to the current framework of the Andalusian Health Service.

## Figures and Tables

**Table 1:** Study variables.

Variable	Variable Type	Definition	Values
Rosendaal TTR	Continuous quantitative	Percentage of time that the INR is within the therapeutic range	0–100
DECAMirt score	Continuous quantitative	Therapeutic adherence measurement tool	0–100
Anticoagulation knowledge test score	Continuous quantitative	Instrument measuring knowledge of oral anticoagulation	0–20
Age	Continuous quantitative	Participant’s age	In years
Gender	Qualitative	Participant’s gender	Man, woman, or nonbinary
Level of education	Qualitative	Level of education of the patients	Primary, Secondary, University, or Other.
Comorbidities	Qualitative	Participant’s comorbidities	Diabetes mellitus, high blood pressure, dyslipidemia, heart disease, or others.
Clinical diagnosis	Qualitative	Inclusion diagnosis for patients on anticoagulants	Atrial fibrillation, valve prostheses, ETEV, thrombophilia…
Place of residence	Qualitative	Place of residence of the participants	Rural or Urban
Years of anticoagulant treatment	Continuous quantitative	Number of years the participant has been on oral VKA therapy	0–20
Vitamin K antagonist	Qualitative	Kind of vitamin K antagonist	Acenocoumarol or Warfarin
Presence of primary caregiver	Qualitative	The patient has a primary caregiver	Yes/No/NA
Mobile app use	Qualitative	The patient uses mobile app	Yes/No/NA
Self-monitoring duration	Continuous quantitative	Number of months the patient has participated in the self-monitoring program	0–60
Weight	Continuous quantitative	Participant’s weight	Weight in kg
Height	Continuous quantitative	Participant’s height	Height in cm

## Data Availability

The authors declare that there is no data available due to the present phase of our study.
